# Monitoring system for the effective instruction based on the semi-automatic evaluation of programs during programming classroom lectures

**DOI:** 10.1186/s41039-015-0019-8

**Published:** 2015-10-13

**Authors:** Satoru Kogure, Riki Nakamura, Kanae Makino, Koichi Yamashita, Tatsuhiro Konishi, Yukihiro Itoh

**Affiliations:** 1 Graduate School of Informatics, Shizuoka University, Hamamatsu, 432-8011 Japan; 2 Faculty of Informatics, Shizuoka University, Hamamatsu, 432-8011 Japan; 3 Faculty of Business Administration, Tokoha University, Hamamatsu, 431-2102 Japan; 4 Shizuoka University, Hamamatsu, 432-8011 Japan

**Keywords:** Programming, Practice monitoring system, Semi-automatic programming evaluation

## Abstract

In this study, we developed a programming practice monitoring system to facilitate teachers to give appropriate instructions to students at the appropriate time during classroom lectures. To help teachers to provide appropriate instruction to learners, we identified parameters that would be useful for teachers during programming exercise in classroom lecture. We constructed a monitoring system with five functions. The system automatically acquired the programs written by students to evaluate their performance, and the teacher can obtain their performance using the five functions. We asked four subjects to test our proposed monitoring system during a simulation of a classroom lecture. The evaluation revealed that the system had a high accuracy in evaluating student programs.

## Background

In the areas of programming and algorithm education, many studies have developed learning support system (Fossati, Eugenio, Brown, and Ohlsson, 2008; Kogure, Okamoto, Noguchi, Konishi, and Itoh, 2012; Malmi et al., 2004; Nakahara, Konishi, Kogure, Noguchi, and Itoh, 2009; Noguchi, Nakahara, Konishi, Kogure, and Itoh, 2010). However, during programming courses for beginners in educational institutions, such as universities, the costs of grading the programs and reports submitted by students are very high. Thus, several automated methods have been developed for the evaluation of student programs, such as LAURA (Adam & Laurent, 1980) and PROUST (Johnson, 1990). We also developed a teacher support environment that focused on supporting teachers during programming education (Kogure, Takatsu, Konishi, and Itoh, 2010). The environment made it easier for teachers to grade programs and text reports. However, the teacher accessed the environment after the classroom lecture finished.

In this study, we address the provision of appropriate instructions from teachers to students during classroom lectures. Lectures mainly involve the provision of exercise by teachers. Teachers also give individual instructions to students while walking around the class and checking what the students are doing. The teacher also provides instructions to the whole class. Typically, the number of teachers is extremely low relative to the high number of students. Therefore, it is very difficult for a teacher to fully check the status of all the students in the classroom. This means that it is necessary for a teacher to obtain answers to questions from each individual student to understand their status fully. Thus, a teacher needs to stop to conduct student exercises to obtain the necessary information. In addition, teachers can only obtain poor quality information in real time. A method is available that uses a clicker-based technique to address these problems (Kennedy and Cutts, 2005). In this method, the teacher gives students a dedicated remote control in advance. When a teacher asks questions during the class, the students answer the questions using a remote control. Thus, the teacher can see the answers immediately. Suppose that a teacher asks students about their progress in a particular exercise. The students can answer the question but the answers are based on their subjective evaluation, and therefore, the answers do not necessarily reflect their actual progress. Thus, the teacher cannot help students who do not correctly understand their progress. On the other hand, Spacco, Hovemeyer, and Pugh present AutoCVS (Spacco, Hovemeyer, and Pugh, 2004), which is Eclipse plug-in for collecting student’s program. Jadud also constructed a programming editor, called BlueJ (Jadud, 2006), for recording a student’s snap-shot on editing their own program. Their systems, although, cannot deal with the possibility of automatically judging and analyzing the student’s program.

Therefore, we propose a method that allows teachers to conduct an objective assessment based on clear criteria. During programming exercises, one of the indicators used as an objective assessment is the program written by the students during the class. In this study, the teacher is supported by the automatic collection of the programs written by the students where the environment automatically analyzes the programs. Thus, a teacher can recognize the progress of students in real time.

Our study aimed to achieve the following:

1. We summarize the information required by teachers during a lecture.
2. We develop a method for extracting the desired information from the programs, which are collected automatically.
3. We build a server that automatically extracts the necessary information from the programs collected.
4. We build a client that presents the extracted information to the teacher.

The programming exercise monitoring system constructed in this study has two components. First, it has an information extraction server that extracts useful information for the teacher. The server collects the students’ programs automatically and extracts the necessary information. Second, it has an instruction support viewer that makes it easier to present the extracted information to the teacher.

The purpose of this study is that the two modules can generate useful information for instruction to all the students. We should discuss whether teachers can give appropriate instruction from the point of view of the teacher’s ability of leadership using two modules, although we do not discuss it in this study.

We assess whether the system could extract the necessary information from the programs collected during real classes. We also have four subjects using the instruction support viewer, and we perform a subjective evaluation by the subjects. A virtual classroom experimental environment is simulated, which collects the programs produced in the real class. The results of our subjective evaluation suggest that collecting the programs produced in classes by students in real time allows the teachers to provide appropriate instructions to students.

## Methods

### Definitions of lecture, exercises, and steps

We define lectures *L*, exercises *E,* and steps *S*. Typically, lectures in higher education institutions are held 15 times or 10 times during a single course. First, we define *L*_i_ as the *i*-th in the course. In each lecture, a teacher will provide exercises to students. Next, we define *E*_j_ as the *j*-th exercise in all lectures. Each exercise that occurs during a lecture may include several small exercises. We refer to these small units as *steps*. Finally, we define *S*_j,k_ as the *k*-th steps of *E*_j_. In addition, a teacher gives the required steps and optional steps to students during the exercise. Thus, we define *isRequired(S*_*j,k*_ *)* as a function that returns *true* if step *S*_j,k_ is required or *false* if step *S*_j,k_ is optional.

### Definitions of the step progress and exercise progress of students

In a class *L*_*i*_, some students will write a program *E*_*j*_ while other students may write a programs *E*_*j’*_ from last week’s lecture *L*_*i–1*_. In addition, some students will be working on the same exercise *E*_*j*_ but on different steps. In addition, a teacher will want to know the progress of each student. We define *isStepFinished(s, j, k)* as a function that returns *true* if student *s* has finished step *S*_*j,k*_ or *false* if he/she has not. Thus, a teacher will know the steps a student has finished. Therefore, we define the step progress s*p(s, j)* in an exercise *E*_*j*_ as the step that student *s* has finished.

In other cases, a teacher may want to know the exercises a student has completed. Thus, we define *isExerciseFinished(s,j)* as a function that returns *true* if student *s* has finished all of required *S*_*j,k*_ or returns *false* if he/she has not finished them. We also define exercise progress *ep(s)* as an exercise *E*_*j*_ that a student *s* is working on.

### Definitions of the student program and standard algorithm

During the class, students will compile the same step in a program many times. The information extraction server automatically collects a student program when a student compiles the program using the wrapped compiler that a teacher gives to the student in advance. A student uses the wrapped compiler when he/she compiles their own program, then the wrapped compiler compiles the student program using the original compiler (e.g. gcc^1^) and sends the program, the student information, and the current time to the information extraction server using secure cp (e.g., scp^2^). We define *p(s,t)* as a program that student *s* compiles at time *t*.

We may want to automatically assess the exercise and step that correspond to *p(s,t)*. Thus, a teacher prepares the correct program for each step in each exercise and translates each program into a standard algorithm *st(j,k)* for step *S*_*j,k*_ in advance. The standard algorithm is represented using Extended PAD (Konishi, Suzuki, Haraikawa, and Itoh, 2007). Our evaluation system can convert a macro operation into various patterns of statements that implement the functions of the macro operation. It is relatively easy to represent various programs as an algorithm with the same function. In addition, Extended PAD can represent various types of arbitrariness. Thus, Extended PAD can use the two extended expressions: “Non-ordering structure” and “Alternative structure.” Our proposed programming exercise monitoring system applied an automatic evaluation module to the student program based on comparing the standard algorithm with Extended PAD expressions derived from the student programs (Konishi et al., 2007).

In addition, if a teacher finds that a program has a distinctive difference from the standard algorithm, he/she may want to search for the same distinctive point in other students’ programs. Therefore, the monitoring system also applies an automatic classification module to student programs, which detects differences from the standard algorithm (Kogure et al., 2010).

## Reused modules

### The assessment module for student programs

We had developed an evaluation system that compared the PAD translated from a student program (s-PAD) with the PAD of a standard algorithm prepared in advance by the teacher (t-PAD), which classified student programs into four categories (Konishi et al., 2007). Our system calculates two matching rates to assess the student programs. First, it calculated the matching rates for s-PAD based on t-PAD. We defined *sar(s, j, k)* as the matching rate so that a number of operations in the s-PAD of student *s* during step *S*_*j,k*_ in exercise *E*_*j*_ corresponding to the operations in the t-PAD divided by the number of all operation in the s-PAD. Second, it calculates the matching rate for the t-PAD based on the s-PAD. We also defined *tar(s, j, k)* as the matching rate so that a number of operations in the t-PAD during step *S*_*j,k*_ in exercise *E*_*j*_ corresponding to the operations in the s-PAD of student *s* divided by the number of all operations in the t-PAD. Table 1 shows the classification types, which were assessed automatically. The information extraction server used modules to calculate *sar(s, j, k)* and *tar(s, j, k)*. We decided classification thresholds shown in Table 1 by heuristic approach based on maximizing classification rates of programs collected in past times from programming course in our university.

**Table 1:** Classifications of student programs

Classification type	Condition required for classification
Perfect	*sar(s,j,k)* = 1 && *tar(s,j,k)* = 1
Excess	*sar(s,j,k)* = 1 && *tar(s,j,k)* > = 0.7
Partial	*sar(s,j,k)* > = 0.75 && *tar(s,j,k)* > = 0.7
No match	*otherwise*

### The module that searched for programs with a particular difference

**Fig. 1:**
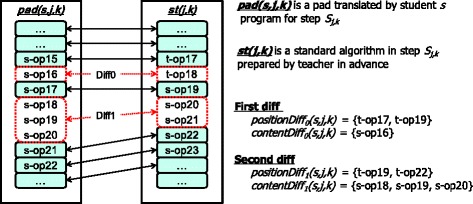
An example of a difference during automatic classification

During the evaluation of the reports and programs submitted by students, a teacher may find a distinctive point (e.g., an error or an additional exercise) in a student’s program. We define *positionDiff*_*i*_ *(s,j,k)* as the range from the previous operation at the beginning of i-th different position in t-PAD to the next operation at the end of the i-th different position in the t-PAD for step *S*_*j,k*_ by student *s*. We also define *contentDiff*_*i*_ *(s,j,k)* as the s-PAD operations at *positionDiff*_*j*_ *(s,j,k)*. For example, Fig. 1 shows *positionDiff()* and *contentDiff()* examples. Thus, a teacher can find programs with the same *positionDiff*_*j*_ *(s,j,k)* or both the same *positionDiff*_*j*_ *(s,j,k)* and the same *contentDiff*_*j*_ *(s,j,k)* using an existing module (Kogure et al., 2010).

## Overview of the programming exercise monitoring environment

**Fig. 2:**
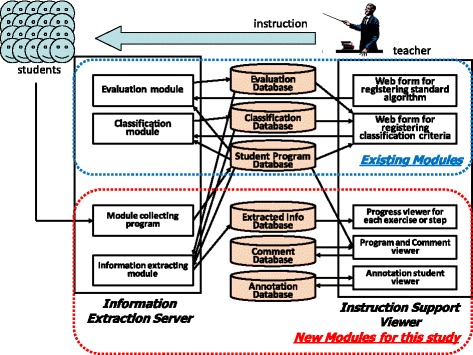
The relationships among the modules and the database

Figure 2 shows the relationships among the modules and the database. The teacher prepared the standard algorithm *st(j,k)* for step *S*_*j,k*_ during exercise *E*_*j*_ in advance. The teacher can create the standard algorithm *st(j,k)* from the correct programs for step S_*j,k*_ or can modify *st(j,k)* using an existing PAD editor.

### Information extraction server

During a lecture, the information extraction server operates in the following steps.

1. A program *p(s,t)* is collected and stored in the database when the server receives the program.
2. The server executes the following operations during each step *S*_*j,k*_. Second item in the first level
  - In the *k-th* step *S*_*j,k*_, the server calculates *sar(s,j,k)* from *p(s,t)* and *st(j,k)* using the existing evaluation module.
  - The server stores information for *p(s,t)*, which is obtained from the evaluation.
3. The server assesses step *S*_*j,k’*_, which corresponds to *p(s,t),* using the Eq. (4.1).
  4.1 $$ {S}_{j,k\hbox{'}}=\underset{S_{j,k}\hbox{'}}{ \arg \max }sar\left(s,j,k\right) $$
4. The system stores the assessment information extracted from the database.

The proposed environment had 11 tables in the database, as shown in Table 2. A teacher prepared the first four data (1–4) in advance. The information extraction server updated the next four data (5–8) in real time during the class. The teacher could register the last three data (9–11) before/during/after the class.

**Table 2:** Database tables

ID	Classification type	Condition required for classification
1	Classes	Information on courses
2	Students	Information on students
3	Registrations	Registration information for a course
4	Exercises	Information on the exercises in a course
5	Personal evaluations	Each students’ evaluation results
6	Personal classifications	Each students’ classification results
7	Class achievements	Summary of the evaluation results for the whole class
8	Class classifications	Summary of the classification results for the whole class
9	Criteria	Classification criteria
10	Comments	Comments tagged by teachers in each programs
11	Attention students	Observable students tagged by a teacher

### Instruction support viewer

The instruction support viewer has five functions, which help the teacher to provide appropriate instructions to students. The five functions are described in the “Function that displays the exercise progress of students in lectures” to “Function that displays the program” sections.

#### Function that displays the exercise progress of students in lectures

If a teacher wants to know the exercise progress of all the students, he/she can use a function that displays the exercise progress ep(s) in a circle graph. The progress ep(s) is calculated using Eq. (4.2).

4.2 $$ ep(s)= latest\left(\left\{{E}_j\Big|sar\left(s,j,k\right) of\kern0.5em  required\kern0.5em {S}_{j,k}\kern0.5em > Threshhold\right\}\right) $$

**Fig. 3:**
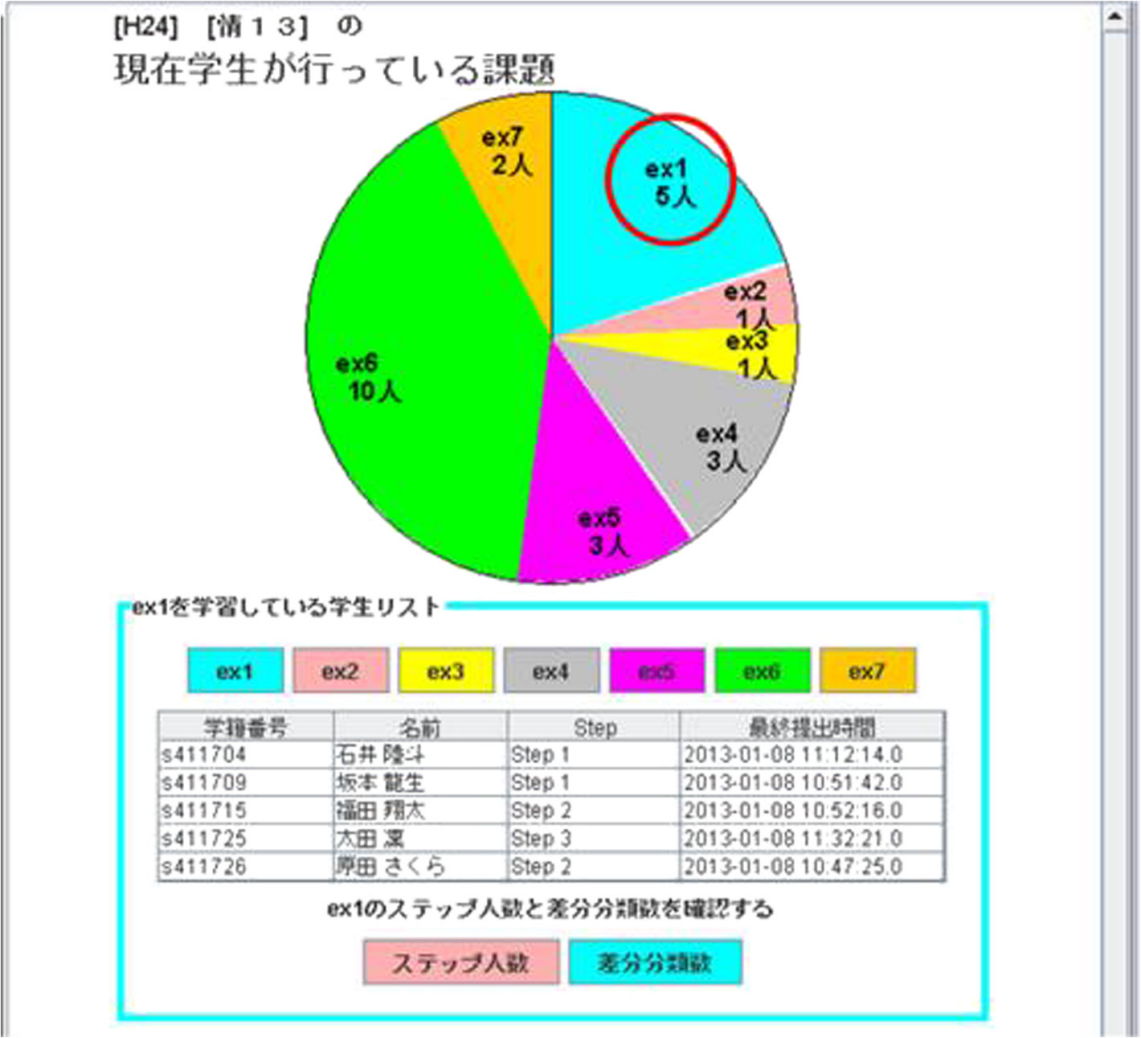
A sample of viewer for exercise progress

Figure 3 shows the viewer for exercise progress. In screenshot, the teacher can understand that five students create a program for exercise ex1. Furthermore, the teacher can show these students’ names.

Thus, the teacher can provide appropriate instruction to the whole class because they can monitor the exercise progress of the class as a whole.

#### Function that displays the step progress of students in exercises

If a teacher wants to know the step progress of all students in an exercise, he/she can use a function that displays the step progress sp(s,j) as a bar graph. The progress sp(s,j), in an exercise Ej is calculated using Eq. (4.3).

4.3 $$ sp\left(s,j\right)=\underset{S_{j,k}\hbox{'}}{ \arg \max }sar\left(s,j,k\right) $$

The teacher can check the change of the step progress over time. Therefore, the teacher can provide detailed instruction on a particular step that most students have been working on for a long time and give initial instruction on the next step to all students.

#### Function that displays the classification results for student programs in an exercise

A teacher may want to know how many students made the same mistake in an exercise. If most students make the same mistake, the teacher may well want to give instruction to the whole class. If a small number of students make the same mistake, the teacher may well want to give specific instruction only to those students.

In this study, a teacher can check each student’s *positionDiff(s,j,k)* and/or *contentDiff(s,j,k)*. If the teacher focuses on a particular *positionDiff(s,j,k)*, the system finds the collection *p(s,t)* that includes the same *positionDiff(s,j,k)* or both the same *positionDiff(s,j,k)* and *contentDiff(s,j,k)*. The instruction support viewer then shows the list of the students whose programs include the same *positionDiff(s,j,k)* or both the same *positionDiff(s,j,k)* and *contentDiff(s,j,k)*.

#### Function that displays a student list tagged with comments

A teacher can tag the student records with comments using the instruction support viewer if the student has unique characteristics (e.g., a student has very high programming skills or his/her attendance is poor). The teacher can then browse the list of students tagged with comments. If there are several teachers or teaching assistants, it is also possible to share information on students using tagged comments.

The instruction support system can display the list of the comments tagged for a particular exercise. The teacher can also read a student’s comments tagged for all exercises if the teacher wants to focus on the student.

#### Function that displays the program

The teacher can examine students’ programs using the viewer if he/she wants to assess the programming progress of those students. The viewer displays the student programs in different windows so that the teacher can compare a student program with those of other students’.

#### Integration of the five functions

A teacher may want to assess the step progress during exercises using the function described in the “Function that displays the step progress of students in exercises section” section while looking at the exercise progress using the function described in the “Function that displays the exercise progress of students in lectures” section. A teacher may also want to tag comments using the function described in the “Function that displays a student list tagged with comments” section while looking at a program produced by a student using the function described in the “Function that displays the program” section. Therefore, the five functions described above should be integrated seamlessly so that several functions can be used together.

## Results

### Evaluation of the information extraction server

During the experimental evaluation of the information extraction server, we focused on two variables: the accuracy of the automatic program evaluation module and the accuracy of step progress analysis using the information extraction server.

The evaluation was conducted as a part of a programming class held in a humanities department. We collected all of the programs compiled by the students in the exercises. The class contained 25 university sophomore students. In this experiment, it was not possible to use the real time transfer program because of a security issue. Thus, we modified the compiler wrapper. The compiler wrapper temporarily stored all of the programs on the student’s PC when they compiled a program. After the class, we manually collected all of the programs that were stored temporarily.

In the lecture, the teacher gave exercise ex7 to all the students. However, some students worked on previous exercises during the lecture. There were seven possible exercises that students worked on, as shown in Table 3. In all exercises, the teacher gave a form of program to all the students. For example, the students have defined the function that returns the minimum value for three numbers in step 3 of ex1.

**Table 3:** Exercises and steps in the exercises

Exercises	Steps
ex1	Step 1, step 2, step 3, step 4 and step 5
ex2	Step 1 and step 2
ex3	Step 1 and step 2
ex4	Step 1 and step 2
ex5	Step 1 and step 2
ex6	Step 1, step 2 and step 3
ex7	Step 1, step 2 and step 3

#### Accuracy of automatic program evaluation module

Table 4 shows the evaluation results for the automatic evaluation module for each of 20 pairs of a student program and the corresponding standard algorithm. We selected randomly 20 pairs in order to eliminate the bias of each student and each exercise. The most important point in this experimental evaluation was the number of false alarms (the cases in which the system’s evaluation was “equal” and the teacher’s evaluation was “not equal”). This is because if there are false alarms, the teacher might overlook mistakes in the student programs. Table 4 shows that the number of false alarms was zero and the overall accuracy was 94.1 % (i.e., (299 + 70)/392). The main reason for the miss (the cases in which the system’s evaluation was “not equal” and the teacher’s evaluation was “equal”) was that the standard algorithm did not cover all the possible alternatives. As mentioned, teachers can describe standard algorithm using alternative representations. However, teachers cannot be prepared in advance all of how to solve an exercise because the most of how to solve one exists.

**Table 4:** Evaluation results for the automatic evaluation module

		System evaluation		Total
		Equal	Not equal	
Teacher’s evaluation	Equal	299 (76.3 %)	23 ( 5.8 %)	322 (82.1 %)
	Not Equal	0 ( 0.0 %)	70 (17.9 %)	70 (17.9 %)
Total		299 (76.3 %)	93 (23.7 %)	392 (100 %)

#### Accuracy of the step progress assessment using the information extraction server

To evaluate the step progress, we manually tagged the correct steps in all the programs. Next, we compared the manually tagged steps with the automatically tagged steps, which were calculated from maximizing *sar(s,j,k)* by *p(s,t)*. Among the 507 programs collected, the system results and manual results were both correct for 381 programs (case A). For 124 programs (case B), the system result was wrong and the manual result was correct. For case A, the accuracy of the step progress assessment was 75.1 %. For case B, the step progress of 24.5 % of the programs was undetectable using the system. This problem occurred because the automatic evaluation module could translate none of the 124 programs into the correct s-PAD due to syntax errors. Since the compiler wrapper stored the student programs when the programs were compiled, hence, the system could not translate those programs with syntax errors into well-formed s-PAD. Thus, we constructed another method that compared programs including syntax errors with the correct program in order to estimate whether the student has conducted any step in each exercise. In this method, the system executed the *diff* command in UNIX (i.e., the command extracting the difference between files). One student program was compared with each of the possible correct programs using the *diff* command. The result with the minimum different lines was adopted as the target of the evaluation. Using this simple method, the system tagged the correct steps in the 124 programs. For the remaining two cases (case C), the students were working on irrelevant programs during a class, and the system correctly judged that those programs involved none of the steps in Table 3.

### Experimental evaluation of the instruction support viewer

To evaluate the instruction support viewer, we registered 25 students in the student database. We performed the experimental evaluation using a virtual environment because our instruction support viewer was a prototype, and we did not want to disadvantage the real students. The virtual environment was a lecture that involved exercise ex7 (as shown in Table 3). The lecture duration was 80 min. In the experimental evaluation, we simulated four situations: 20 min, 40 min, 60 min, and 80 min (the end of the lecture) after the beginning of the lecture. There were four subjects in the evaluation. One was a teacher who lectured for each exercise described in Table 3. The other three subjects had experience as teaching assistants in a department of informatics. We asked the four subjects to emulate the teacher’s actions in these situations using the instruction support viewer. We asked them to obtain the necessary information for answering those questions in Table 5 by using the instruction support viewer.

**Table 5:** The questions provided to the subjects in each situation

	Question
20 min	How many students worked on exercise ex7? How many students worked on each step in exercise ex7?
	Who worked on ex1 or ex2?
40 min	Who worked the fastest on the exercise? Check the student program and tag the student.
	Who had poor programming skills? You may use the search function in the student annotation database.
60 min	Who was the student who needed special attention? What was the step progress of the student?
	How many students worked on step1 in ex5? Assess whether you needed to provide instruction to all of the students or specific students.
80 min	How many students finished exercise ex7? Assess whether there is a need to teach a catch-up class. If so, what would be the exercise in the catch-up class?

All the four subjects had no trouble in using the instruction support viewer. The subjects also gave appropriate answers to those questions in Table 5 by using the viewer. We also conducted a subjective evaluation about merits/demerits of using the viewer through a questionnaire. We received positive evaluations for each of the five functions and some comments for further improvements.

## Discussion

### Function that displays the exercise progress of students in lectures

In the evaluation experiment, subjects actively use the function that displays the exercise progress of students on pie chart. This result indicates that this function has enough features necessary to view the progress of the class. However, we obtain the subjective evaluation that operation method is not intuitive. That is, it is not possible to also click on pie chart and view the details of each. It is necessary to improve the interface to allow user to operate intuitively.

### Function that displays the step progress of students in exercises

By looking at the bar graph for step progress, it is possible to grasp the step progress of students of particular exercise in more detail. If almost all the students were working the same exercise, the step progress could be considered of value. In the evaluation of this experiment, we have assumed that the majority of students are promoted to exercise ex7. However, only 2 of 24 students reached exercises ex7 actually. Therefore, the effective use of this function was not so much.

### Function that displays the classification results for student programs in an exercise

By using this feature, teachers can grasp immediately the number of students who wrote the similar program to particular program. However, we obtain a subjective assessment that it is difficult to understand how to use the function. It is necessary to improve the interface to allow users to operate intuitively.

### Function that displays a student list tagged with comments

For teachers, ability to leave comments is very beneficial. Further, even when multiple teachers lecture, there is an advantage that teachers can share detailed information. However, we obtain a subjective evaluation that it is difficult to grasp the comment one by one by increasing the comments. It is necessary to add the ability to search for comments and to improve the view ability of the comments.

### Function that displays the program

The teacher can confirm a program written by a student and can check a comment written by the teacher for him/her using the function. The teacher also can use the function by clicking the student name on viewer when he/she uses other four functions. Furthermore, it is possible to introduce the instruction to individual student. However, we obtain a subjective evaluation that it is difficult to handle interfaces when comparing a student’s program with other student’s program or correct program.

### Others

From the subjects’ questionnaire after the experiment, we obtained the opinion that they want to use the system on mobile devices. We had assumed that the teacher use the system on the stationary PC. However, it is not possible to effectively use the system during an individual guidance.

## Conclusions

We developed a programming exercise monitoring system to facilitate teachers to give appropriate instructions to students at the right time during classroom lectures. Our programming exercise monitoring system has five functions. The system collects the programs written by students automatically. Teachers can assess the collected programs using the integrated five functions. We collected 507 programs during an actual programming exercise in a classroom lecture. We asked four subjects to use our proposed monitoring system in a simulated classroom lecture. The evaluation revealed that the system had high accuracy in evaluating student programs and that the five functions were useful in real classroom settings.

In the future, we will construct new monitoring system for individual instruction. The teacher can use this system on mobile device, and teachers and teaching assistants will share the information of an individual instruction and will use the stored information for other student’s individual instruction.

## Footnotes

1 The GNU Compiler Collection (GCC) is a compiler system produced by the GNU Project supporting various programming languages.

2 The Secure Copy (SCP) is a means of securely transferring computer files between a local host and a remote host or between two remote hosts.

## References

[CR1] Adam A, Laurent JP (1980). LAURA, a system to debug student programs. Artif Intell.

[CR2] Fossati D, Eugenio BD, Brown C, Ohlsson S (2008). Learning linked lists: Experiments with the iList System.

[CR3] Jadud MC (2006). Methods and tools for exploring novice compilation behaviour.

[CR4] Johnson WL (1990). Understanding and debugging novice programs. Artif Intell.

[CR5] Kennedy GE, Cutts QI (2005). The association between students’ use of an electronic voting system and their learning outcomes. J Comput Assist Learn.

[CR6] Kogure S, Okamoto M, Noguchi Y, Konishi T, Itoh Y (2012). Adapting guidance and externalization support features to program and algorithm learning support environment.

[CR7] Kogure S, Takatsu H, Konishi T, Itoh Y (2010). Development and evaluation of learning support system based on automatic classification of students’ programs according to difference from standard algorithm.

[CR8] Konishi T, Suzuki H, Haraikawa T, Itoh Y (2007). Three phase self-reviewing system. Knowledge Management for Educational Innovation.

[CR9] Malmi L, Karavirta V, Korhonen A, Nikander J, Seppala O, Silvasti P (2004). Visual algorithm simulation exercise system with automatic assessment: TRAKLA2. Informatics in Education.

[CR10] Nakahara T, Konishi T, Kogure S, Noguchi Y, Itoh Y (2009). Learning environment for algorithm and programming where learners operate objects in a domain world using GUI.

[CR11] Noguchi Y, Nakahara T, Konishi T, Kogure S, Itoh Y (2010). Construction of a learning environment for algorithm and programming where learners operate objects in a domain world. International Journal of Knowledge and Web Intelligence.

[CR12] Spacco J, Hovemeyer D, Pugh W (2004). An Eclipse-based course project snapshot and submission system.

